# Supplementary material for: MinD proteins regulate CetZ1 localization in Haloferax volcanii
Source: Front Microbiol. 2024 Nov 22;15:1474697. doi: 10.3389/fmicb.2024.1474697 (PMC11621097; doi:10.3389/fmicb.2024.1474697)
Supplement: Supplementary file 1 [file Data_Sheet_1.pdf]

Supplementary Material

MinD proteins regulate CetZ1 localisation in *Haloferax volcanii*

Hannah J. Brown[1] and Iain G. Duggin[1]

[1]Australian Institute for Microbiology and Infection, University of Technology Sydney, Ultimo, NSW 2007, Australia.

Contents:

Legends for Supplementary Videos 1-4

Supplementary Figures

1. Polar localisation of CetZ1-mTq2 can be partially restored by complementation of minD2 deletion.
2. CetZ1-mTq2 has no effect on motility or rod-shape in *minD* deletion backgrounds.
3. Cell circularity and motility analysis of chromosomal and plasmid-expressed minD2 Walker mutants.

Supplementary Tables

1. Strains used in this study
2. Plasmids used in this study
3. Oligonucleotides used in this study

Legends for Supplementary Videos:

1. Z-stack of 3D-SIM reconstructions showing CetZ1-mTq2 localization in WT (top), Δ*minD2* (middle), and Δ*minD4* (bottom). The first frame is the bottom-most Z-position, and the last frame is the top-most Z-position.
2. 3D render of 3D-SIM images showing CetZ1-mTq2 localization in the WT genetic background.
3. 3D render of 3D-SIM images showing CetZ1-mTq2 localization in the Δ*minD2* genetic background.
4. 3D render of 3D-SIM images showing CetZ1-mTq2 localization in the Δ*minD4* genetic background.

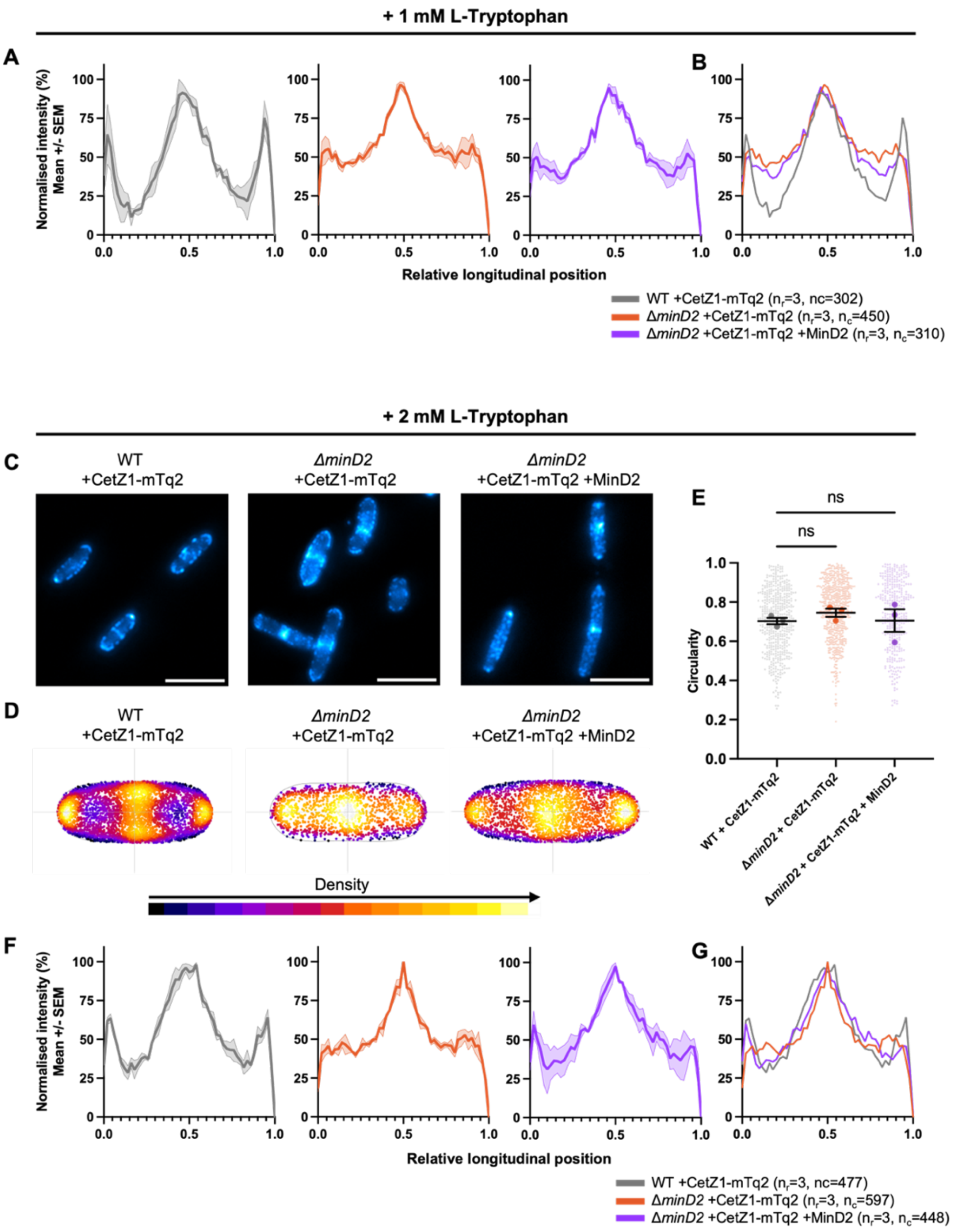

Figure S1. Polar localisation of CetZ1-mTq2 can be partially restored by complementation of *minD2* deletion. CetZ1-mTq2 was produced in the wildtype (H26) and Δ*minD2* backgrounds from pHVID135. MinD2 and CetZ1-mTq2 were simultaneously produced from pHJB72, containing the *cetZ1-mTq2* ORF immediately downstream from the *p.tna* promotor, followed by *minD2* in a tandem configuration. Three independent culture replicates (n_c) were carried out for each strain, and cells were sampled for imaging from Hv-Cab medium supplemented with 1 mM **(A-B)** or 2 mM **(C-F)** L-Tryptophan inducer, at an approximate OD_{600} of 0.2. **A)** The median fluorescence intensity of CetZ1-mTq2 along the normalised long axis of each cell. Data were normalised (0-100%) for each culture replicate and plotted to show the mean (bold line) and standard error (shading) of all culture replicates. **B)** Overlay of the mean data in **A.** The number of culture replicates (n_r) and total number of individual cells analysed is indicated in the key. **C)** Fluorescence microscopy of CetZ1-mTq2 in the wildtype background, Δ*minD2* background, and Δ*minD2* background when co-produced with MinD2 for complementation. **D)** Heatmaps representing the position and number (heatmap density) of detected CetZ1-mTq2 fluorescence peak intensities (foci) along the long (vertical) and lateral (horizontal) axes of all cells combined. **E)** Superplots showing cell circularity measurements of each strain. Small individual points represent individual cells, large individual points represent mean cell circularity values of culture replicates. One-way ANOVA was used as a statistical test, ns=not significant. **F** Is as per **A**, and **G** as per **B,** but with 2 mM L-Tryptophan.

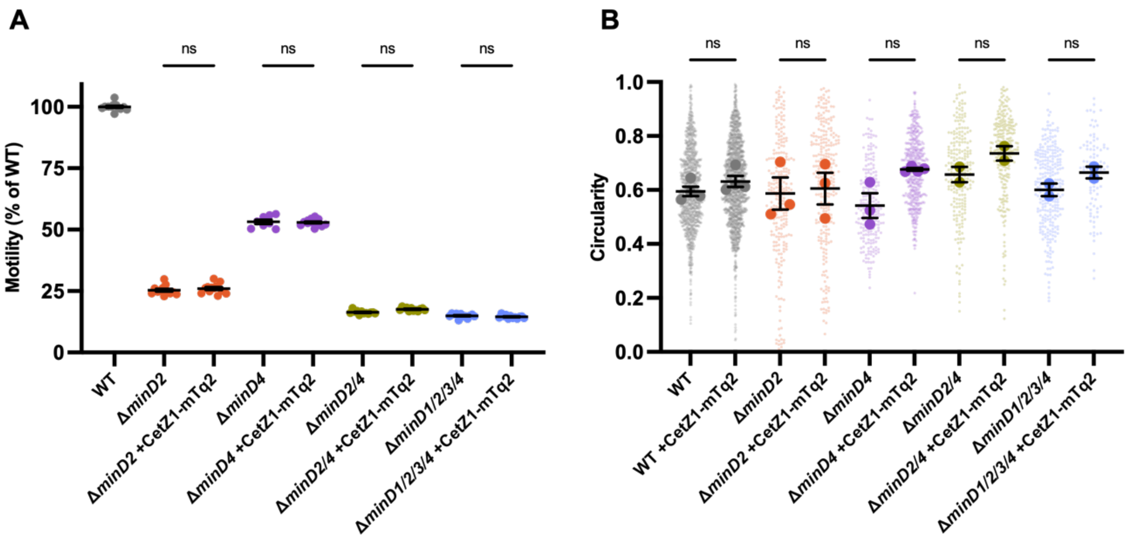

Figure S2. CetZ1-mTq2 has no effect on motility or rod-shape in *minD* deletion backgrounds. Wildtype and *minD* deletion strains containing the empty vector pTA962 or pHVID135 for expression of CetZ1-mTq2 were subjected to soft-agar motility assays and cell shape quantification. The relevant data from Fig. 1 and 4 are included here for reference. **A)** Quantification of halo diameter from Hv-Cab + 1 mM L-Tryptophan soft-agar (0.25%) motility assays. Individual points represent biological replicates. Mean and standard error are shown. **B)** Superplot showing cell circularity of cells grown in Hv-Cab medium with 1 mM L-Tryptophan and sampled at an approximate OD_{600} of 0.2. Large data points represent mean circularity of one culture replicate, small data points represent individual cells from all culture replicates for the indicated strain. Mean and standard error of culture replicate means is shown. The number of individual cells measured from pooled biological replicates for *minD* deletion strains containing pTA962 are detailed in the legend of Fig. 1, and for remaining strains are as follows: **(B)** WT + CetZ1-mTq2, n=1341; Δ*minD2* + CetZ1-mTq2, n=271; Δ*minD4* + CetZ1-mTq2, n=432; Δ*minD2/4* + CetZ1-mTq2, n=251; Δ*minD1/2/3/4* + CetZ1-mTq2, n=97. One-way ANOVA was used as a statistical test (n=3), ns=not significant.

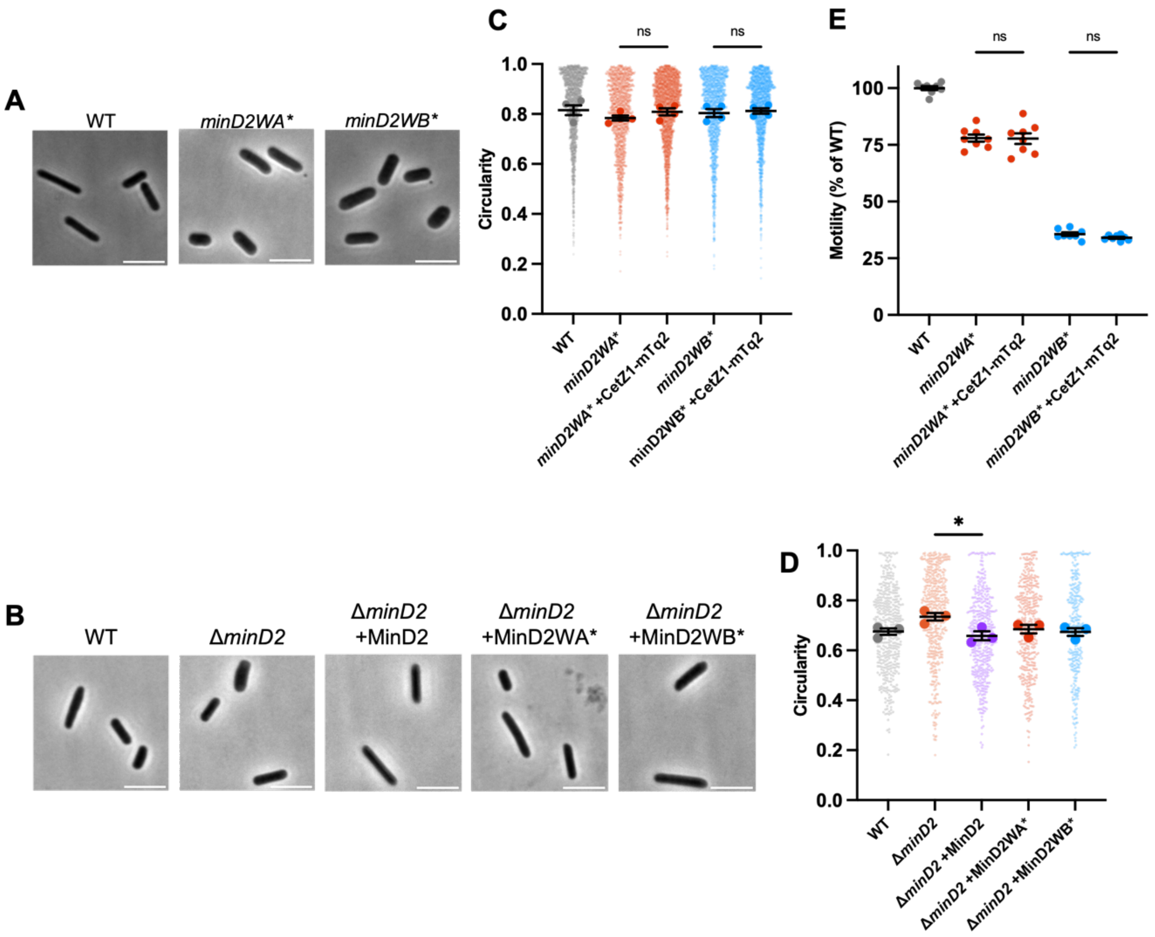

Figure S3. Cell circularity and motility analysis of chromosomal and plasmid-expressed *minD2* Walker mutants. A) Representative phase-contrast microscopy images of *minD2* chromosomal walker mutants containing pTA962 and **B)** Δ*minD2* containing pHJB63-65 for expression of *minD2*, *minD2WA**, and *minD2WB**, respectively, were grown to an approximate OD_{600} of 0.2 in HvCab medium supplemented with 1 mM L-Tryptophan (scale bars are 5 µm) . **C)** Superplot showing cell circularity quantification of cells in **A** and *minD2* chromosomal Walker mutants with CetZ1-mTq2 (from Fig. 5a). Large datapoints represent mean circularity of all cells within one culture replicate. Small data points represent individual cells from all culture replicates in the indicated strain. Mean and standard deviation of culture replicate means is shown. The total number of cells analysed from pooled biological replicates is as follows: WT + pTA962, n=1851; minD2WA* + pTA962, n=1488; minD2WA* + CetZ1-mTq2, n=2334; minD2WB* + pTA962, n=1649; minD2WB* + CetZ1-mTq2, n=1830; **D)** As in **C**, but for strains shown in **B.** The total number of cells analysed from pooled biological replicates is as follows: WT + pTA962, n=446; Δ*minD2* + pTA962, n=500; Δ*minD2* +MinD2, n=456; Δ*minD2* +MinD2WA*, n=428; Δ*minD2* +MinD2WB*, n=319. **E)** Quantification of halo diameter from Hv-Cab + 1 mM L-Tryptophan soft-agar (0.25%) motility assays for strains in **A/C.** Individual points represent biological replicates. Data from Fig. 4c is included here for reference. Mean and standard error are shown. In **C-E,** one-way ANOVA was used as a statistical test (n=3 for **C** and **D**). Only significant comparisons are shown. *p<0.05, ns=not significant.

Table S1. Strains used in this study

Strain	Description	Source
ID621 (H26)	Wildtype strain, cured of pHV2 and *ΔpyrE2*	[1]
HTQ228 (Δ*minD2*)	H26 with deletion of *minD2* (HVO_0595)	[2]
HTQ218 (Δ*minD4*)	H26 with deletion of *minD4* (HVO_0322)	[2]
HTQ241 (Δ*minD2/4*)	H26 with deletion of *minD2* (HVO_0595) and *minD4* (HVO_0322)	[2]
HTQ262 (Δ*minD1/2/3/4*)	H26 with deletion of *minD1* (HVO_0225), *minD2* (HVO_0595), *minD3* (HVO_1634), and *minD4* (HVO_0322)	[2]
ID810 (*minD2WA**)	H26 with a point mutation (K16A) within the walker A motif of *minD2*	This study
ID811 (*minD2WB**)	H26 with a point mutation (D117A) within the walker B motif of *minD2*	This study

Table S2. Plasmids used in this study

Plasmid	Description	Source
pTA962	Expression vector containing *p.tna* promotor for *H. volcanii* and an *E. coli* shuttle plasmid	[3]
pHVID135	For expression of CetZ1-G-mTurquoise2 fusion protein	[4]
pTA131_minD2WA	pTA131-based vector containing flanking sequences and Walker A mutation for minD2. Used to generate the chromosomal Walker A mutant minD2 strain *minD2WA**.	This study, Genscript
pTA131_minD2WB	pTA131-based vector containing flanking sequences and Walker B mutation for minD2. Used to generate the chromosomal Walker B mutant minD2 strain *minD2WB**.	This study, Genscript
pHJB63	For expression of *minD2* from pTA962 (between NdeI and BamHI).	This study
pHJB64	For expression of *minD2WA** from pTA962 (between NdeI and BamHI).	This study
pHJB65	For expression of *minD2WB** from pTA962 (between NdeI and BamHI).	This study
pHJB72	For dual expression of *cetZ1-mTq2* and *minD2* under the control of the same *p.tna* promotor. The ORF for *minD2* is cloned between NheI and NotI restriction sites of pHVID135, putting it in the second position behind *cetZ1-mTq2*.	This study

Table S3. Oligonucleotides used in this study

Name	Sequence (5'-3')	Use
MinD2_USflank_extF	CGACGTCGATTGC GTCCT	For amplification of *minD2* to confirm chromosomal point mutation of Walker A and Walker B motifs.
MinD2_DSflank_extR	GCTTTTGAACCGC TCTGCGA	For amplification of *minD2* to confirm chromosomal point mutation of Walker A and Walker B motifs.
NdeI-MinD2_F	GGCGGCCATATG GTCGAGGCGTTC GCC	For amplification of *minD2* to incorporate an NdeI site at the 5' end of the product. Used to generate pHJB63-65.
MinD2_BamHI_R	GGCGGCGGATCC TCATATTCGCTCG GGGACG	For amplification of *minD2* to incorporate a BamHI site at the 3' end of the product. Used to generate pHJB63-65.
XbaI_MinD2_F	GGCGGCTCTAGA ATGGTCGAGGCGT TCGCC	For amplification of *minD2* to incorporate a XbaI site at the 5' end of the product. Used to generate pHJB72.
T3	GCAATTAACCCTC ACTAAAGG	For amplification of *minD2* for the generation of pHJB72.

References:

1. Allers T, Ngo H-P, Mevarech M, Lloyd RG: **Development of additional selectable markers for the halophilic archaeon *Haloferax volcanii* based on the *leuB* and *trpA* genes**. *Applied and environmental microbiology* 2004, **70**(2):943-953.
2. Nußbaum P, Ithurbide S, Walsh JC, Patro M, Delpech F, Rodriguez-Franco M, Curmi PM, Duggin IG, Quax TE, Albers S-V: **An oscillating MinD protein determines the cellular positioning of the motility machinery in archaea**. *Current Biology* 2020, **30**(24):4956-4972. e4954.
3. Allers T, Barak S, Liddell S, Wardell K, Mevarech M: **Improved strains and plasmid vectors for conditional overexpression of His-tagged proteins in *Haloferax volcanii***. *Applied and environmental microbiology* 2010, **76**(6):1759-1769.
4. Ithurbide S, de Silva RT, Brown HJ, Shinde V, Duggin IG: **A vector system for single and tandem expression of cloned genes and multi-colour fluorescent tagging in *Haloferax volcanii***. *Microbiology* 2024, **170**(5).
